# Use of Cytokine Mix-, Imiquimod-, and Serum-Induced Monoculture and Lipopolysaccharide- and Interferon Gamma-Treated Co-Culture to Establish In Vitro Psoriasis-like Inflammation Models

**DOI:** 10.3390/cells10112985

**Published:** 2021-11-02

**Authors:** Katarzyna Bocheńska, Marta Moskot, Magdalena Gabig-Cimińska

**Affiliations:** 1 Department of Medical Biology and Genetics, University of Gdańsk, Wita Stwosza 59, 80-308 Gdańsk, Poland; katarzyna.bochenska@phdstud.ug.edu.pl; 2 Laboratory of Molecular Biology of Human Skin Diseases, Institute of Biochemistry and Biophysics, Polish Academy of Sciences, Kładki 24, 80-822 Gdańsk, Poland

**Keywords:** cell-based models, in vitro cultures, skin, epidermal keratinocytes, monocytes, psoriasis

## Abstract

Psoriasis (Ps), commonly perceived as a skin and joint disorder, has a complex basis and results from disturbances in the sophisticated network between skin and the immune system. This makes it difficult to properly depict the complete pathomechanism on an in vitro scale. Deciphering the complicated or even subtle modulation of intra- and intercellular factors, assisted by the implementation of in vitro human skin models, may provide the opportunity to dissect the disease background step by step. In addition to reconstructed artificial skin substitutes, which mimic the native physiological context, in vitro models are conducive to the broad “3 Rs” philosophy (reduce, refine, and replace) and represent important tools for basic and applied skin research. To meet the need for a more comprehensive in vitro Ps model, a set of various experimental conditions was applied in this study. The selection of in vitro treatment that mimicked the Ps phenotype was illustrated by analyses of discriminating biomarker genes involved in the pathogenesis of the disease, i.e., keratinocyte differentiation markers, antimicrobial peptides, chemokines, and proliferation markers. This resulted in a reproducible protocol for the use of the primary skin keratinocyte (pKC) monoculture treated with a cytokine cocktail (5MIX, i.e., interleukin (IL) 1 alpha (IL-1α), IL-17A, IL-22, oncostatin M (OSM), and tumour necrosis factor alpha (TNF-α)) at a calcium (Ca^2+^) concentration (i.e., 2 mM) in an applied medium, which best mirrored the in vitro Ps-like inflammatory model. In addition, based on waste skin material, the method has the potential for extensive experimentation, both in detailed molecular studies and preclinical tests.

## 1. Introduction

Characterisation of the molecular and cellular basis of human diseases is essential for the development of personalised and more effective methods of diagnosis, prevention, and therapy in contemporary biomedical sciences. Standardised, pre-clinical research models, refined so that the original conditions of the experiment are repeatable and thus reproduced in detail by other teams of researchers, are challenging and difficult to elaborate. These models are essential for fundamental molecular investigations and contribute to a better understanding of the potential causes of human diseases [1]. Moreover, pre-clinical research is an important stage in drug development. Tests to assess the effectiveness and safety of new substances with potential therapeutic effects and determine the pharmacological, pharmacokinetic, and toxic properties of the tested compounds are performed in vitro on cell models or in vivo on animal representations [2].

Psoriasis (Ps) is a chronic systemic inflammatory disease that affects 2–3% of the world’s population. It is frequently associated with some pathologies, namely cardiovascular dysfunctions, respiratory insufficiency and gastrointestinal problems [3]. In recent years, significant progress has been made in elucidating the molecular mechanisms of this type of dermatosis. However, many of its aspects remain unexplained, including epithelial and immune system disturbances, autoimmune-based inflammation, the relationship between skin and systemic factors, and the role of genetic and environmental influences on disease onset, progression, and response to treatment [4,5]. It usually manifests as oval plaques covered with silvery scales, resulting from epidermal hyperproliferation (evidenced by the Ki67 marker) that is characterised by the disturbed maturation of keratinocytes and incomplete keratosis with the retention of cell nuclei in the stratum corneum (parakeratosis) [6]. At the root of the disease, initiating events are the interplay between environmental and genetic factors. Triggers such as mechanical trauma or bacterial infections initiate a cascade of events that include the formation of DNA-LL37 complexes, activation of plasmacytoid dendritic cells, and secretion of proinflammatory cytokines [7]. The stimulated myeloid dendritic cells migrate to the draining lymph nodes and cause the differentiation of naïve T cells into effector cells, such as T helper 1 or 17 cells (Th1 or Th17) or T cytotoxic 1 or 17 cells (Tc1 or Tc17). Immune cells (mainly neutrophils) expressing the CXCL1, CXCL2, and CXCL8 receptors migrate to the skin along a chemokine gradient [8,9]. Key processes during disease persistence are the presentation of putative autoantigens to T lymphocytes and the release of interleukin 23 (IL-23) by skin dendritic cells, the production of proinflammatory mediators, such as IL-17A, IL-17F, and IL-22, by Th17 and Tc17 lymphocytes, and interferon gamma (IFN-γ) and tumour necrosis factor alpha (TNF-α) by Th1 and Tc1 cells [10,11]. These mediators act on keratinocytes, leading to their activation and proliferation as well as the production of antimicrobial peptides (e.g., LL-37 cathelicidin and β-defensins), chemokines (e.g., CXCL9 via CXCL11 and CCL20), and S100 proteins (e.g., S100A7 via S100A9) [12,13]. The circle is closed by the simultaneous action of keratinocytes, fibroblasts, immune cells, and endothelial cells, which are involved in tissue reorganisation by the progression of chronic inflammation (mediated by phosphoinositide 3-kinase/mammalian target of rapamycin kinase (PI3K/mTOR) pathway) [14]. Therefore, the affected skin exhibits abnormal maturation, manifested by a mutation or decrease in the gene expression of part of early and terminal differentiation markers (*FLG*, *KRT1*, *KRT5*, *KRT10*, *KRT14*, and *LOR*) and an increase in the activity of suprabasal cell markers (*IVL*, *KRT6*, and *KRT16*) [15,16].

Depending on the type of experiments conducted, two-dimensional (2D) in vitro Ps models are based on homogeneous cultures of primary cells isolated from human skin (keratinocytes, fibroblasts, and Langerhans cells) and cell lines (genetically modified, immortalised cells) [17]. The selection of Ps phenotype-inducing agents depends on the type of research and the expected effect. Typically, such experiments are based on the addition of pro-inflammatory cytokines (mainly interleukin (IL) 1 alpha (IL-1α), IL-1 beta (IL-1β), IL-6, IL-17A, IL-17F, IL-22, oncostatin M (OSM), or TNF-α) or their combination in the culture medium [18,19]. Another model approach is based on the co-culture of different types of cells (keratinocytes with fibroblasts or keratinocytes with immune system cells). Cells may remain in direct contact or can be separated by a pore-size barrier. The pores are large enough to ensure the exchange of substances produced by cells, which is essential for crosstalk between different types of cells when modelling the disease in vitro [20].

In this study, various in vitro models were evaluated: monocultures of human adult low calcium temperature keratinocytes (HaCaT) cell line and primary keratinocytes (pKCs), stimulated with a cytokine mix (5MIX, i.e., IL-1α, IL-17A, IL-22, OSM, and TNF-α) or imiquimod (IMQ), HaCaTs stimulated with Ps patients’ serum, and co-culture of HaCaTs with human leukaemia monocytic cell line (THP-1) treated with phorbol 12-myristate 13-acetate (PMA), lipopolysaccharide (LPS), and IFN-γ. The induction of the Ps phenotype was evidenced by analysis of the expression of strictly selected marker genes involved in the process of disease pathogenesis, i.e., *CCL20*, *CXCL1*, *CXCL2*, *CXCL8*, *DEFB4*, *FLG*, *IVL*, *KRT1*, *KRT5*, *KRT6*, *KRT10*, *KRT14*, *KRT16*, *LOR*, *MKI67*, *PI3*, *S100A7*, and *S100A9*.

## 2. Materials and Methods

### 2.1. Cell Lines and Maintenance of Mono- and Co-Cultures

Human adult low calcium temperature keratinocytes (HaCaTs) were obtained from CLS Cell Lines Service GmbH (Eppelheim, Germany). Cells from passage 39−42 were seeded at a density 5 × 10^4^ cells/cm^2^ in Dulbecco’s modified Eagle’s medium (DMEM, Gibco, Thermo Fisher Scientific, Waltham, MA, USA) with 4500 mg/L glucose and 2 mM L-glutamine (Gibco, Thermo Fisher Scientific, Waltham, MA, USA), supplemented with 1% (*v*/*v*) antibiotic/antimycotic (Sigma-Aldrich, Saint Louis, MO, USA), and 10% (*v*/*v*) foetal bovine serum (FBS) (Gibco, Thermo Fisher Scientific, Waltham, MA, USA). Cells were cultured in T-flasks at 37 °C in 5% CO_2_ humidified atmosphere until reaching 70–80% confluence prior to experimentation and then passaged using trypsin/ethylenediaminetetraacetic acid (EDTA) (0.25% (*w/v*) solution; Gibco, Thermo Fisher Scientific, Waltham, MA, USA).

pKCs were isolated from histologically normal skin punches of three healthy donors and then utilised for three independent cultures (i.e., *n* = 3 biological replicates). The study was assembled within the framework of our collaboration with the Department of Dermatology, Venerology, and Allergology at the Medical University of Gdańsk. Signed informed consent was obtained from all subjects under protocols approved by the Independent BioEthics Committee of the Medical University of Gdańsk (NKBBN/161-634/2018). The epidermis was dissociated from the dermis by incubation with 10 U/mL Dispase II (Sigma-Aldrich, Saint Louis, MO, USA) at 4 °C for 16 h. The epidermis was washed 3 times with phosphate-buffered saline (PBS) (Gibco, Thermo Fisher Scientific, Waltham, MA, USA), cut into smaller pieces, and treated with 0.05% trypsin/EDTA at 37 °C to obtain a single cell suspension. The number of viable cells was determined using a Scepter 2.0 Cell Counter (Merck Millipore, Burlington, MA, USA). Cells were seeded at a density of 5 × 10^6^ cells/cm^2^ in DMEM with 4500 mg/L glucose and 2 mM L-glutamine, supplemented with 1% (*v*/*v*) Antibiotic-Antimycotic (A/A) solution (Sigma-Aldrich, Saint Louis, MO, USA), and 10% (*v*/*v*) FBS at 37 °C in a humidified atmosphere with 5% CO_2_. After 24 h, the medium was changed to EpiGRO™ medium (Merck Millipore, Burlington, MA, USA) supplemented with L-glutamine, EpiFactor P, epinephrine, rh transforming growth factor alpha (TGF-α), hydrocortisone hemisuccinate, rh Insulin, apo-transferrin (Merck Millipore, Burlington, MA, USA), and 1% (*v*/*v*) A/A solution. Cells were cultured in T-flasks to 70–80% confluence prior to experimentation and then passaged using trypsin/EDTA (0.05% (*w/v*) solution).

For keratinocyte and immunological cells, co-culture of HaCaT and THP-1 was introduced. THP-1 monocytes were acquired from CLS Cell Lines Service GmbH. They were routinely maintained in RPMI 1640 medium supplemented with 2 mM glutamine, 10% (*v*/*v*) FBS bovine serum, and 1% (*v*/*v*) A/A solution at 37 °C in a humidified atmosphere with 5% CO_2_. The number and viability of cells was estimated with Tuerk solution and a Neubauer haemocytometer to maintain cell culture density at 2–8 × 10^5^ cells/mL. The medium was replaced every third day, and the culture was split 1:5 when it reached 1 × 10^6^ cells/mL.

Keratinocyte and/or immunological cells (i.e., monocultures of HaCaT or pKC sourced from donors, and co-culture of HaCaT and THP-1) were used for three independent culture replicates (*n* = 3) and applied in further analyses.

### 2.2. Stimulation to Psoriasis (Ps)-Like Inflammation Status

#### 2.2.1. Stimulation with a Pro-Inflammatory Mix of Cytokines

For stimulation, HaCaT and pKC cell cultures were carried out until 70% confluence was achieved. After this time, keratinocytes were enzymatically digested using trypsin/EDTA (0.25 or 0.05% (*w*/*v*)) for HaCaT and pKC, respectively, and seeded on 6-well plates at a density of 5 × 10^3^ cells/cm^2^ in serum-free medium (SFM, Gibco, Thermo Fisher Scientific, Waltham, MA, USA) with the addition of 30 μg/mL bovine pituitary extract (BPE), 0.2 ng/mL epidermal growth factor (EGF; Gibco, Thermo Fisher Scientific, Waltham, MA, USA), and 1% (*v*/*v*) A/A solution to a final concentration. Following a 24 h incubation period, the medium was changed to SFM without growth supplements for a further 16 h of incubation. Subsequently, cells were activated with a cytokine mix (5MIX, i.e., interleukin (IL) 1 alpha (IL-1α), IL-17A, IL-22, oncostatin M (OSM), and tumour necrosis factor alpha (TNF-α), 2 ng/mL each; Abcam, Cambridge, United Kingdom) for a further 24 h or left non-activated as a control. As an additional panel to induce keratinocyte differentiation, activated or non-activated cells with the addition of 2 mM calcium ions were also tested.

#### 2.2.2. Stimulation with Imiquimod (IMQ)

HaCaT and pKC cells were cultured until 70% confluence was achieved, and then they were trypsinised (trypsin/EDTA, 0.25 or 0.05% (*w*/*v*), respectively) and seeded on 6-well plates at a density of 5 × 10^3^ cells/cm^2^. Cells were maintained in SFM, with the addition of 30 μg/mL BPE, 0.2 ng/mL EGF, and 1% (*v*/*v*) A/A solution to a final concentration. Following a 24 h incubation period, the medium was changed to SFM without growth supplements, with the addition of IMQ (Sigma-Aldrich, Saint Louis, MO, USA) for a further 24 h or left untreated as a control.

#### 2.2.3. Stimulation with Serum

HaCaT cells were grown to 70% confluency and then plated into 6-well plates using trypsin/EDTA (0.25% *w/v*) and maintained in SFM with the addition of 30 μg/mL BPE, 0.2 ng/mL EGF, and 1% (*v*/*v*) A/A solution to a final concentration. After 24 h, the medium was changed to SFM supplemented with serum from Ps patients and healthy people in three concentrations (*v*/*v*): 1, 5, and 10%. Each culture was terminated at three time points: 24, 48, and 72 h.

#### 2.2.4. Stimulation with Phorbol 12-Myristate 13-Acetate (PMA), Lipopolysaccharide (LPS), and Interferon Gamma (IFN-γ)

The transwell cell culture system was used for indirect psoriatic stimulation. Prior to co-culture, 6 × 10^6^ THP-1 cells were transferred to 3 mL RPMI 1640 medium supplemented with 2 mM glutamine, 10% (*v*/*v*) FBS, and 1% (*v*/*v*) A/A solution in cell culture membrane inserts (Falcon Permeable Support for 6 Well Plate with 1.0 μm Transparent PET Membrane) placed in 6-well plates with an additional 2 mL RPMI medium. Monocyte-to-macrophage M1 stimulation was induced with 6 h incubation of THP-1 with PMA (30 ng/mL; Sigma-Aldrich, Saint Louis, MO, USA). Further cells were incubated for 66 h with IFN-γ (5 or 10 ng/mL; Sigma-Aldrich, Saint Louis, MO, USA) and LPS (10 or 50 ng/mL; Sigma-Aldrich, Saint Louis, MO, USA) used alone and in combination. Control cells were cultivated in RPMI 1640 alone.

The degree of inflammatory stimulation was estimated indirectly with the Lactate-GloTM assay (Promega, Madison, WI USA), a bioluminescent assay for the detection of L-lactate in biological samples, according to the manufacturer’s instructions. Stimulated inflammatory and cancer cells are characterised by a higher glucose metabolism, and lactate is a major product of this process. Conditions with the highest THP-1 lactate secretion were selected for further HaCaT stimulation.

For co-cultivation studies, HaCaT cells (4 × 10^5^) grown in 6-well plates in RPMI 1640 medium supplemented with 2 mM glutamine, 10% (*v*/*v*) FBS, and 1% (*v*/*v*) A/A solution were washed twice with PBS (Sigma-Aldrich, Saint Louis, MO, USA). After PMA/LPS/IFN-γ stimulation, cell culture inserts with THP-1 cells were washed twice with PBS and then placed on top of the HaCaT cells to avoid physical contact of the different cell lines. HaCaT and THP-1 co-cultures were maintained in freshly added RPMI 1640 medium for the next 24 h at 37 °C in a humidified atmosphere with 5% CO_2_. The HaCaT monolayer in RPMI 1640 was the control in this experiment.

#### 2.2.5. Cell Viability Assay

To estimate cell proliferation, HaCaT or pKC cells were seeded (8 × 10^3^ cells per well) in 96-well plates and grown overnight on SFM without growth supplements at 37 °C in a humidified atmosphere with 5% CO_2_. IMQ (Sigma-Aldrich, Saint Louis, MO, USA) was then added to the wells to achieve a final concentration ranging from 10 to 250 μM. Control cells were left untreated. All dilutions were tested in triplicate for 24 h, 48 h, and 7 days. Subsequently, 3-(4,5-Dimethylthiazol-2-yl)-2,5-diphenyltetrazolium bromide (MTT) dye (5 mg/mL; Sigma-Aldrich, Saint Louis, MO, USA) was added to each well, and plates were incubated at 37 °C in a humidified atmosphere with 5% CO_2_ for 4 h. Then, 100 µL DMSO (≥99.5%) was added to solubilise the formazan product. The optical density of each well was measured using a Victor3™ plate reader (PerkinElmer, Waltham, MA, USA), with excitation at 490 nm and emission at 520 nm. Percentage calculations were performed for the controls, assuming a value of 100%. The half maximal inhibitory concentration (IC50) was calculated by reading the IMQ concentration (μM) at which 50% of the cells remained viable relative to the control.

#### 2.2.6. RNA Handling and Reverse Transcription

The total RNA was extracted from cells using a High Pure RNA Isolation Kit (Roche Applied Science, Penzberg, Germany) following the manufacturer’s instructions. The concentration and purity of RNA were determined by the Quant-iT™ RiboGreen™ RNA Assay Kit (Invitrogen, CA, USA) and agarose gel electrophoresis, respectively. cDNA synthesis was performed using the Transcriptor First-Strand cDNA Synthesis Kit (Roche Applied Science, Penzberg, Germany).

#### 2.2.7. Real-Time Quantitative Reverse Transcriptase-Polymerase Chain Reaction (Real-Time qRT-PCR)

Real-time qRT-PCR analysis was carried out on a LightCycler^®^ 480 Instrument II (Roche Applied Science, Penzberg, Germany) using LightCycler^®^ 480 Probes Master Mix (Roche Applied Science, Penzberg, Germany). All data represent averaged values ± standard deviation from three independent culture replicates (*n* = 3), with three technical repeats each (*N* = 3). Expression values were normalised against the TATA-binding protein (*TBP*, Hs00427620_m1) reference gene. The TaqMan probes used were as follows: *CCL20* (Hs00355476_m1), *CXCL1* (Hs00236937_m1), *CXCL2* (Hs00601975_m1), *CXCL8* (Hs00174103_m1), *DEFB4* (Hs00823638_m1), *FLG* (Hs00856927_g1), *IVL* (Hs00846307_s1), *KRT1* (Hs00196158_m1), *KRT5* (Hs00361185_m1), *KRT6* (Hs04194231_s1), *KRT10* (Hs00166289_m1), *KRT14* (Hs00265033_m1), *KRT16* (Hs00373910_g1), *LOR* (Hs01894962_s1), *MKI67* (Hs00606991_m1), *PI3* (Hs00160066_m1), *S100A7* (Hs01923188_u1), and *S100A9* (Hs00610058_m1; Thermo Fisher Scientific, Waltham, MA, USA).

#### 2.2.8. Gene Expression Omnibus (GEO) Analyses

Gene expression profiles of 6 datasets of in vitro cell cultures of keratinocytes and 7 datasets of lesional skin biopsies and paired non-lesional and/or normal tissues were obtained from the GEO database of the National Center of Biotechnology Information (NCBI; dataset numbers of in vitro keratinocyte cultures: GDS2081, GDS2611, GDS2732, GDS3011, GDS4598, and GDS4599; and dataset numbers of skin samples: GDS2518, GDS3539, GDS4600, GDS4602, GDS4606, GDS5392, and GDS5420). The selected GEO dataset was normalised and converted with the base-2 logarithm. Differentially expressed genes (DEGs) were identified based on the calculation of the relative quantification ratio between the two groups (Ps/control culture cells; paired lesional/non-lesional and lesional/normal tissues).

## 3. Results

### 3.1. Designation of Gene Markers

To determine an in vitro method to generate Ps skin-like inflammatory model candidates, a screening of Ps skin-specific marker genes was of principal significance. Therefore, the transcription profile of a Ps-like cutaneous in vitro model was established with the use of 18 genes designated on the basis of our earlier study [21] and literature reports (Table 1) on monocultures of human adult low calcium temperature keratinocytes (HaCaTs) or pKCs, stimulated either with a combination of cytokines (5MIX, i.e., interleukin (IL) 1 alpha (IL-1α), IL-17A, IL-22, oncostatin M (OSM), and tumour necrosis factor alpha (TNF-α), 2 ng/mL each), imiquimod (IMQ), or serum, and in co-cultures of keratinocytes and human monocytes (THP-1) treated with phorbol 12-myristate 13-acetate (PMA), lipopolysaccharide (LPS), and interferon gamma (IFN-γ) for Ps-like inflammation stimulation, constituting the basis of this study. In most of the tested cell cultures and experimental conditions, a total of 18 carefully selected discriminating marker genes were tested, and among them, there were genes coding for 9 keratinocyte differentiation markers (i.e., *IVL*, *FLG*, *KRT1*, *KRT5*, *KRT6*, *KRT10*, *KRT14*, *KRT16*, and *LOR*), 4 antimicrobial peptides (i.e., *DEFB4*, *PI3*, *S100A7*, and *S100A9*), 4 chemokines (*CCL20*, *CXCL1*, *CXCL2*, and *CXCL8*), and 1 proliferation marker (i.e., *MKI67*; Table 1). Additionallyreference gene, *TBP*, were used in this research.

### 3.2. Cytokine Mix-Stimulated Psoriasis (Ps)-Like Inflammation Response in HaCaT and pKC

**Figure 1:**
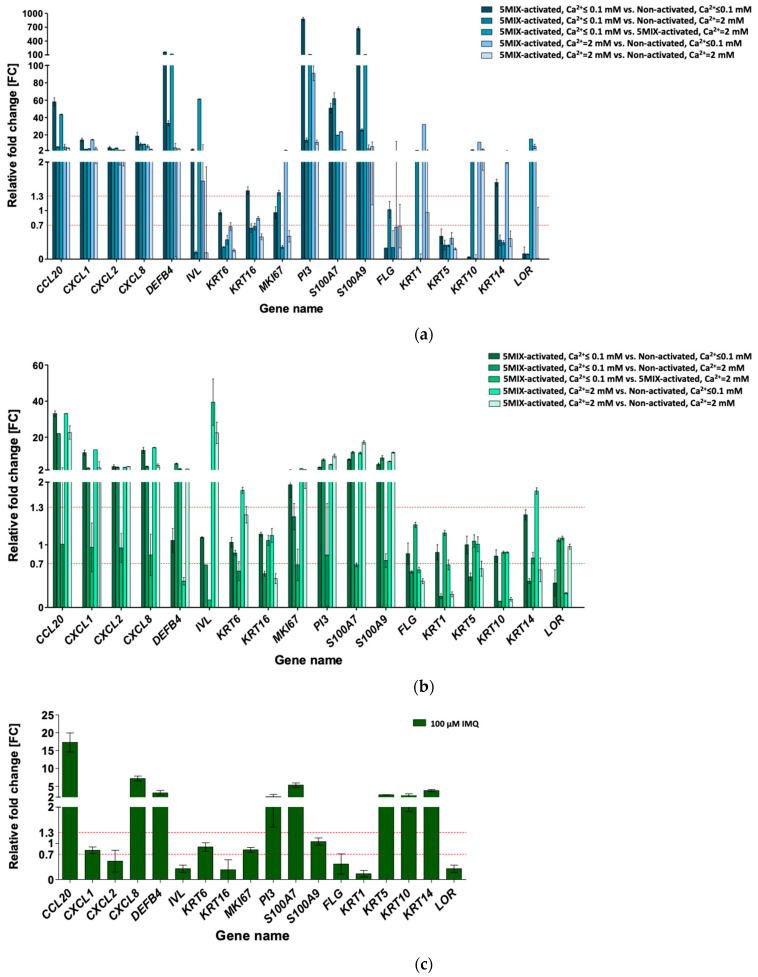

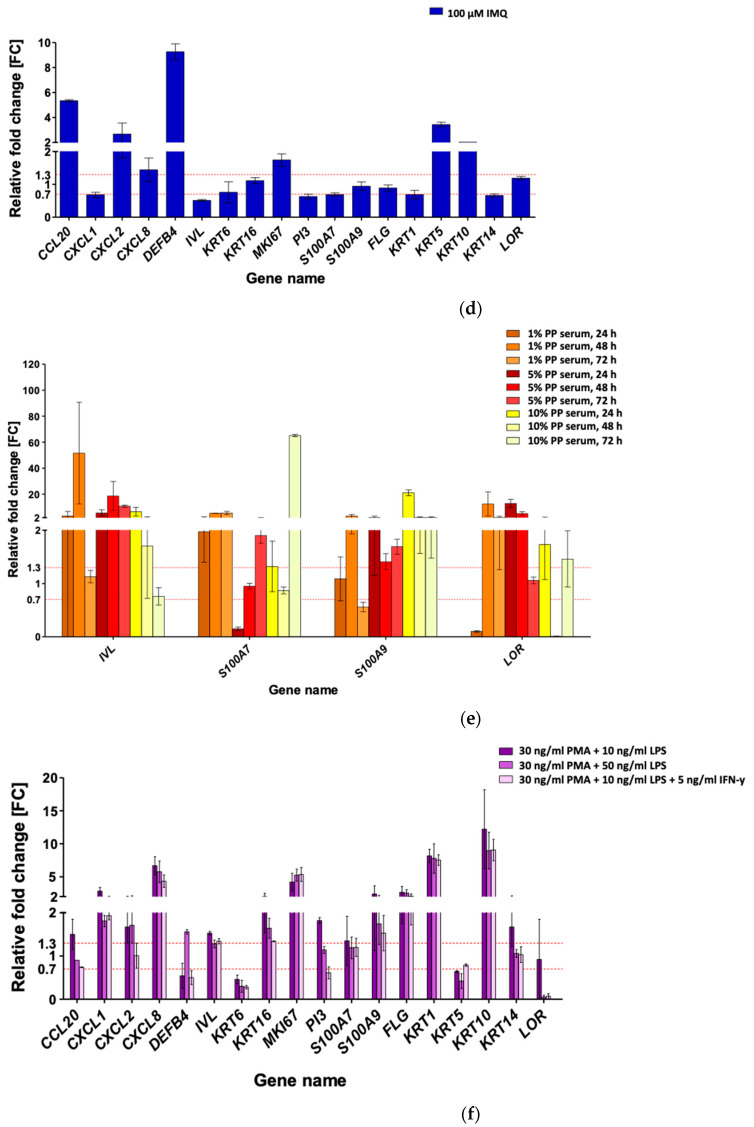
Alterations in mRNA levels (fold change (FC): increase/decrease marked with a horizontal red dotted line, i.e., FC ≥ 1.3 and ≤ 0.7) of selected genes in in vitro psoriasis (Ps)-like inflammation models with cytokine mix (5MIX) at calcium (Ca^2+^) ≤ 0.1 mM or Ca^2+^ = 2 mM (**a**) for HaCaT and (**b**) for pKC; imiquimod (IMQ) (**c**) for HaCaT and (**d**) for pKC; and serum-induced monoculture (**e**) for HaCaT of keratinocytes, and phorbol 12-myristate 13-acetate (PMA), lipopolysaccharide (LPS), and interferon gamma (IFN-γ) co-culture of HaCaT and THP-1 (**f**). The real-time qRT-PCR data represent averaged values ± standard deviation from three independent culture replicates (*n =* 3), with three technical repeats each (*N* = 3), and denote significant differences for tested samples, with respect to the reference gene *TBP*, of a constant expression level implemented for the analysis. Determination of potential candidates for endogenous control as reference genes for real-time qRT-PCR was assessed using the commercially available RealTime ready Human Reference Gene Panel. In addition, analyses of the normalised gene expression data were performed in Prism (GraphPad).

At 0.1 mM calcium (Ca^2+^), the addition of 5MIX inhibited HaCaT differentiation compared to the non-treated control (i.e., 5MIX-activated, Ca^2+^ ≤ 0.1 mM vs. non-activated, Ca^2+^ ≤ 0.1 mM; Figure 1a). *FLG*, *KRT1*, *KRT5*, *KRT10*, and *LOR* exhibited decreased expression (0.23, 0.01, 0.47, 0.04, and 0.1-fold reduction, respectively). As a mimicry of crosstalk between lymphocytes and keratinocytes, 5MIX strongly induced the expression of all eight tested proinflammatory genes, whose products are ordinarily secreted by epidermal cells, i.e., *CCL20*, *CXCL1*, *CXCL2*, *CXCL8*, *DEFB4*, *PI3*, *S100A7*, and *S100A9* (58.17-, 14.19-, 5.34-, 18.63-, 160.06-, 875.38-, 50.89-, and 671.41-fold increase, respectively), when compared to the non-activated cells when both were maintained in Ca^2+^ ≤ 0.1 mM medium. HaCaTs do not fully reflect the response to extracellular calcium intake because of spontaneous immortalisation. However, in our study, the addition of calcium ions to the 5MIX-stimulated cell culture (5MIX-activated, Ca^2+^ = 2 mM vs. non-activated, Ca^2+^ ≤ 0.1 mM) caused a reduction from 2 to more than 30-fold in the expression of five of the eight tested proinflammatory genes (i.e., *CCL20*, *CXCL2*, *CXCL8*, *DEFB4*, and *PI3*) compared to the cells maintained with low calcium ions (5MIX-activated, Ca^2+^ ≤ 0.1 mM vs. non-activated, Ca^2+^ ≤ 0.1 mM), with a simultaneous enhancement in the expression of four of six tested keratinocyte late differentiation markers (*KRT1*, *KRT10*, *KRT14*, and *LOR*). In contrast, the addition of calcium ions to the culture of non-activated cells induced the expression of selected proinflammatory genes. In the non-activated cells at Ca^2+^ = 2 mM, the relative fold change of *DEFB4* and *PI3* was lower than that in the non-activated cells at Ca^2+^ ≤ 0.1 mM (both compared to 5MIX-activated at Ca^2+^ ≤ 0.1 mM culture; fold change (FC) of *DEFB4*: from 33.3 to 160.1, respectively, and FC of *PI3*: from 13.9 to 875.4, respectively), approaching the value noted for 5MIX activated in the Ca^2+^ ≤ 0.1 mM culture. Calcium ions are important for inducing keratinocyte differentiation, which was evident in the increased expression of some differentiation markers tested here (i.e., *KRT1*, *KRT10*, *KRT14*, and *LOR*) for 5MIX activated at Ca^2+^ = 2 mM compared to the non-activated cells at Ca^2+^ ≤ 0.1 mM (32.12-, 11.63-, 1.99-, and 6.77-fold increase, respectively). Nevertheless, in the case of the HaCaT cell line, the results most reflecting the Ps phenotype (and therefore, in accordance with the gene expression profiles shown in Table 1) were obtained for the keratinocytes activated with 5MIX at Ca^2+^ ≤ 0.1 mM in relation to non-activated cells at Ca^2+^ ≤ 0.1 mM, which was confirmed by modulation of 15 of 18 genes (i.e., 10 up- and 5 downregulated; 83% of analysed transcripts).

The addition of 5MIX alone did not inhibit the differentiation of pKCs. However, this did occur in the HaCaT cell line (5MIX-activated, Ca^2+^ ≤ 0.1 mM vs. non-activated, Ca^2+^ ≤ 0.1 mM) and affected the induction of all eight proinflammatory genes tested, i.e., *CCL20*, *CXCL1*, *CXCL2*, *CXCL8*, *MKI67*, *PI3*, *S100A7*, and *S100A9* (FC of 33.06, 11.55, 3.88, 12.85, 1.96, 3.48, 7.79, and 5.04, respectively), as positive feedback loops only triggered immune activation (Figure 1b). However, 5MIX alone inhibited the terminal differentiation of cultures grown at high concentrations of calcium ions (5MIX-activated, Ca^2+^ = 2 mM vs. non-activated, Ca^2+^ = 2 mM), which was evident in the decreased expression of *FLG*, *KRT1*, *KRT5*, *KRT10*, and *KRT14* (FC of 0.42, 0.21, 0.62, 0.13, and 0.60, respectively) in pKCs. No significant effect of calcium ions on 5MIX-treated cells was noted, i.e., the same expression pattern of almost all studied genes (except for *IVL* and *KRT16*) was observed for 5MIX-activated pKCs maintained in Ca^2+^ ≤ 0.1 mM or Ca^2+^ = 2 mM vs. non-activated cells upheld in Ca^2+^ = 2 mM. Conversely, the addition of calcium ions to the cultures of non-activated pKCs was necessary to obtain the phenotype of differentiated keratinocytes, as was evidenced by alterations in FC values of *FLG*, *KRT1*, *KRT5*, *KRT10*, and *KRT14* in pKCs treated with 5MIX-activated at Ca^2+^ ≤ 0.1 mM compared to non-activated cells at Ca^2+^ = 2 mM. In the case of pKCs, there were two experimental environments, 5MIX at Ca^2+^ ≤ 0.1 mM and non-activated at Ca^2+^ = 2 mM, with modulation of 14 of 18 genes (i.e., 9 up- and 5 downregulated; 78% of analysed transcripts), of which only *IVL* and *KRT16* differed from the predictable result. 5MIX at Ca^2+^ = 2 mM, with modulation of 16 of 18 genes (i.e., 11 up- and 5 downregulated; 89% of analysed transcripts) compared to the non-activated cells at Ca^2+^ = 2 mM, best reflected the phenotype of the disease (Table 1) because they both resulted in a Ps-specific profile.

### 3.3. Imiquimod (IMQ) as Ps-Like Inflammation Factor in HaCaT and pKC

IMQ, a ligand of toll-like receptors 7 and 8 (TLR 7/8) on plasmacytoid dendritic cells (pDCs), has been reported to induce Ps-resembling skin lesions. In addition to its immune-inducing properties, several studies have shown its direct effect on selected keratinocyte signalling pathways [35]. To validate the model, HaCaT cells were treated with 100 μM IMQ to induce Ps-like inflammation (Figure 1c). The addition of IMQ to HaCaT cultures resulted in increased expression of the *CCL20* (FC: 17.28), *CXCL8* (FC: 7.19), *DEFB4* (FC: 3.18), *PI3* (FC: 2.09), and *S100A7* (FC: 5.36) markers related to the early inflammatory response. However, *CXCL2*, *IVL*, *KRT5, KRT10, KRT14*, and *KRT16* expression significantly deviated from the Ps gene activity profile (Table 1). This indicates that the use of IMQ alone provides an incomplete picture of this disease, as only 8 of 18 genes (i.e., 5 up- and 3 downregulated; 44% of analysed transcripts) were modulated in HaCaTs, while the pattern of other genes’ expression was much different from that of the Ps mode (Table 1).

As with HaCaT, a weak impact on the induction of the Ps phenotype (Table 1) was also observed in pKCs after stimulation with IMQ (Figure 1d). Only 5 (i.e., *CCL20*, *CXCL2*, *CXCL8*, *DEFB4*, and *MKI67*) of 12 genes considered upregulated (Table 1) had an increased expression. With reference to the genes whose expression was reduced in the Ps phenotype (Table 1), in pKC keratinocytes, *KRT1*, and *KRT14* were downregulated. For the remaining markers, FC was either unchanged or was significantly different from those typical for the Ps mode. In summary, of the 18 genes, 5 genes were upregulated and 2 were downregulated (39% of analysed transcripts) in pKC cells, while the expression pattern did not match those that were predetermined. This indicates the insufficiency of the tested IMQ-activated in vitro keratinocyte model.

### 3.4. Serum-Mediated Ps-Like Inflammation Modulation in HaCaT

To obtain a broader view of the transcriptional changes in tested Ps markers, HaCaTs were incubated with serum derived from patients with Ps (PP serum) and healthy donors as a control (NN serum) to mimic the microenvironment of disease (Figure 1e). Cells were stimulated with 1, 5, and 10% serum mix obtained from five patients and five healthy donors for 24, 48, and 72 h, and four genes in total (i.e., *IVL*, *LOR*, *S100A7*, and *S100A9*) were profiled. The maximum serum action on *IVL* expression was obtained after 48 h of incubation with PP serum (FC of 51.63, 18.62, and 1.70 at 1, 5, and 10% PP serum in relation to NN serum, respectively). A similar, although lower, *IVL* expression was noted for the 24 h treatment with PP serum (FC of 3.21, 5.59, and 6.61 at 1, 5, and 10% PP serum in relation to NN serum, respectively). In HaCaT cultures terminated after 72 h of serum incubation, no changes with respect to *IVL* mRNA were noted for 1 and 10% PP serum compared to the NN serum. Nevertheless, a 10-fold increase was visible in HaCaTs maintained with 5% serum for 72 h when compared to the NN serum control for this gene. In addition to immunological factors, serum, as a source of calcium, could be a possible cause of the unexpected increase in *LOR* transcription in HaCaTs, independent of the concentration and time of PP serum stimulation, except for the following cultures: 24 h with 1% and 48 h with 10% PP serum compared to NN serum (FC of 0.10 and 0.01, respectively). Furthermore, Ps patients’ serum treatment had a greater effect on the induction of expression of the S100 family genes. For *S100A7*, increased expression was observed, especially for 72 h of incubation with PP serum compared to NN serum treatment (FC of 5.48, 1.90, and 65.12 at 1, 5, and 10% PP serum, respectively). In addition, the strongest stimulation effect of the Ps patients’ serum on the *S100A9* mRNA level occurred in the 24 h treatment (FC of 2.08 and 21.06 at 5 and 10% PP serum, respectively, when compared to NN control), followed by the 48 h treatment (FC of 3.19, 1.41, and 2.08 at 1, 5, and 10% PP serum, respectively, compared to the NN serum) and 72 h treatment with 5 and 10% PP serum compared to NN control (FC of 1.69 and 2.08, respectively). Such differentiated data could have resulted from the size and composition in relation to the health condition of the control group (being a source of the NN serum). Analysis of the composition of control sera originating from NN individuals, made using the MAGPIX^®^ System, revealed the presence of proinflammatory cytokines, i.e., IL-8 and TNF-α, which could have affected the results (unpublished data). Nevertheless, based on the analysis of the expression of four genes, it can be concluded that incubation with 1% serum for 24 h is sufficient and most appropriate to obtain the Ps phenotype, since three out of four tested genes (*IVL, S100A7,* and *LOR*) were modulated in a Ps-specific mode.

### 3.5. Phorbol 12-Myristate 13-Acetate (PMA), Lipopolysaccharide (LPS), and Interferon-Gamma (IFN-γ) for Ps-Like Inflammation Stimulation in HaCaT with THP-1

Across several monocyte-to-macrophage differentiation protocols, based on the literature [36,37] and our preliminary experiments [38], conditions with 30 ng/mL PMA were chosen to induce monocyte-to-macrophage differentiation. To enhance inflammatory stimulation, LPS (10 and 50 ng/mL) and IFN-γ (5 ng/mL) were added to monocyte cultures of THP-1.

The most essential differences in gene expression were noticed in cultures treated with 30 ng/mL PMA and 50 ng/mL LPS for *LOR*, whose activity was lowered 20-fold compared to the control and almost 15-fold when cells were treated with 30 ng

PMA, 10 ng/mL LPS, and 5 ng/m IFN-γ (Figure 1f). Only culture conditions with 50 ng/mL LPS distinguished the profile of *DEFB4* to more Ps, with its upregulated expression. HaCaT and THP-1 co-cultivation, preceded by monocyte stimulation either with PMA and 10 ng/mL LPS, PMA and 50 ng/mL LPS, or PMA, 10 ng/mL LPS, and 5 ng/mL IFN-γ resulted in a significant upregulation of *CXCL8* (FC of 6.67, 5.75, and 4.34, respectively) and *MKI67* (FC of 4.19, 5.26, and 5.35, respectively) compared to the control, following the gene modulation profile in Ps (Table 1).

When co-culture of HaCaTs was performed with THP-1 cells activated with 30 ng/mL PMA, 10 ng/mL LPS, and 5 ng/m IFN-γ, only 7 of 18 genes (i.e., 6 up- and 1 downregulated; 39% of analysed transcripts) revealed altered expression (Figure 1f). For THP-1 treatment with 30 ng/mL PMA and 50 ng/mL LPS, modulation of 10 of 18 genes (i.e., 8 up- and 2 downregulated; 56% of analysed transcripts) was observed. The results most reflecting the Ps phenotype (in accordance with the gene expression profiles shown in Table 1) were obtained for HaCaT cells cultivated with THP-1 stimulated with 30 ng/mL PMA and 10 ng/mL LPS, with 11 of 18 genes (i.e., 10 up- and 1 downregulated; 61% of analysed transcripts) showing results typical for Ps.

### 3.6. Statement of In Vitro Culture and Skin Biopsies Data from GEO in Relation to Our Results

**Figure 2:**
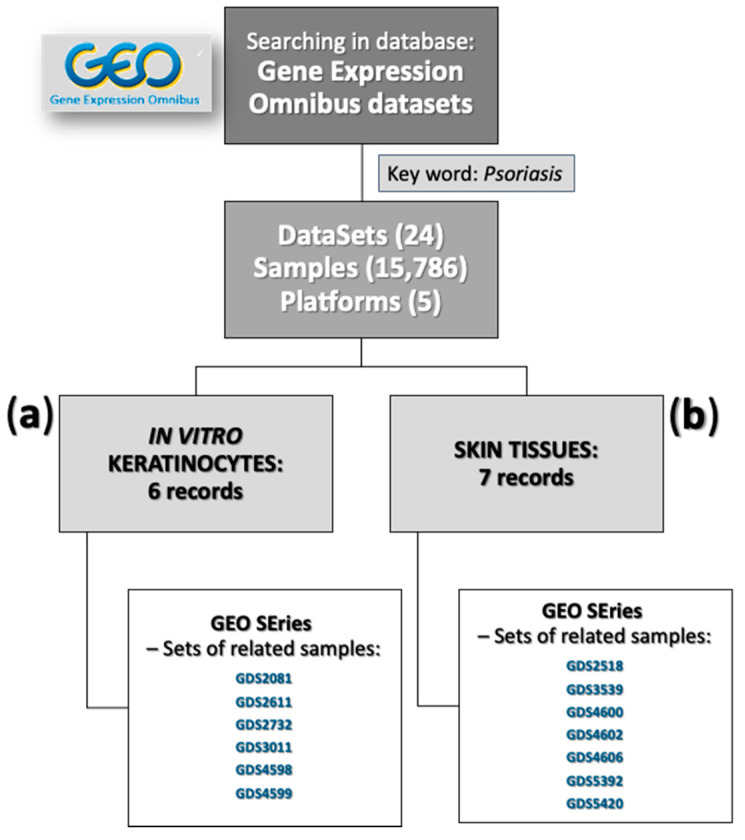
Study pipeline that represents the identification of registers referring to the Gene Expression Omnibus (GEO) profiles of psoriasis (Ps) in vitro cell culture of keratinocytes (**a**); and Ps lesional (PP) vs. non-lesional (PN), PP vs. normal (**b**).

To confirm the relevance of the in vitro models, the expression of selected marker genes was compared with Gene Expression Omnibus (GEO) database profiles downloaded from six datasets for in vitro cell culture of keratinocytes and seven datasets for skin biopsies (Figure 2).

When referring to the six datasets (i.e., GDS2081, GDS2611, GDS2732, GDS3011, GDS4598, and GDS4599 microarray datasets; Figure 2a) and all other series in the public databases for in vitro cell culture of keratinocytes, there were no data deposited for investigations testing 5MIX of cytokines, as used in our studies. Based on the GDS2611 database for subfamily IL-20 analyses concerning keratinocyte in vitro cultures, an increase in the expression of only *CCL20*, *CXCL1*, *CXCL2*, *CXCL8*, *S100A7*, and *S100A9* was noted after stimulation with IL-1β, IFN-γ, IL-19, IL-20, IL-22, or IL-24. The expression of the other 11 tested Ps markers remained unchanged, pointing to the need for the synergistic action of 5MIX of cytokines, which was implemented in our studies to obtain the full phenotype of the disease in vitro. Treatment of keratinocytes with IL-1 (GDS3011) affected the FC value of most of the 18 genes studied in this analysis. However, the obtained profile was not consistent with the Ps phenotype (Table 1), especially considering the predicted downregulated genes (*KRT1*, *KRT5*, *KRT14*, and *LOR*), whose expression was unchanged or increased in the GDS3011 study. Other procedures of cell activation (GDS2081, GDS2732, GDS4598, and GDS4599) did not lead to the development of an appropriate in vitro model characterised by the lack of cell differentiation and the induction of an early inflammatory response in keratinocytes, which was demonstrated by certain profiles of modulation for Ps marker genes.

When referring to the seven datasets for skin biopsies (i.e., GDS2518, GDS3539, GDS4600, GDS4602, GDS4606, GDS5392, and GDS5420 microarray datasets), a change in the expression of a significantly greater number of markers tested in Ps skin was noted in this study (Figure 2b). To eliminate the false results, only lesional skin samples with the corresponding non-lesional skin samples and lesional vs. the average of the group normal skin samples were analysed in our study. Total pairs of lesional (PP) and non-lesional (PN), PP and normal (NN), and PN and NN skin samples were selected from all seven microarray datasets. For the majority of the 18 genes selected in this study and verified in Ps skin samples positioned in the GEO database, the FC value was consistent with the expected increased expression characteristic for the Ps phenotype. However, the FC values of *FLG*, *KRT1*, *KRT5*, *KRT10*, *KRT14*, and *LOR* were unchanged or increased, and not lowered. Similar results were obtained for the PP vs. NN skin sample comparison (GDS2518, GDS3539, and GDS4602). Differences in the expression profiles, mainly for genes downregulated in Ps (Table 1), may be the result of the composition of skin tissue samples and the presence of other types of cells (i.e., fibroblasts, melanocytes, Langerhans cells, macrophages, adipocytes, mast cells, Schwann cells, and other immune cells), affecting the mean FC value.

## 4. Discussion

In vivo human trials are clearly the most reliable means of determining the effect of drugs; however, due to the enormous costs, they are used only to evaluate final products. Live animal experimentation is thus commonplace, and numerous approaches have been used. Generally, animals are the mainstay of anti-inflammatory activity determinations prior to testing in humans. Such approaches are relatively straightforward but come at significant costs due to husbandry and licensing [39]. Tests undeniably cause discomfort to the subject animals, which by convention would be sacrificed at the end of the procedures and are therefore not aligned with the current drive for the “3 Rs” (reduction, refinement, and replacement). In vitro disease models based on cell cultures are in this light a promising alternative to animal testing. Cultured human cells have been widely used to model and determine the anti-inflammatory effects of novel drugs’ effects on skin. Moreover, in vitro skin models have been used to provide information on altered regulation of gene expression (e.g., *IL-17A*, *IL-17F*, and *IL-23* in Ps) and hence to evaluate the effects of cytokine antagonists, which is the first step when developing a biological therapy [40,41]. Unfortunately, not every result obtained in this type of research translates into the actual therapeutic effect of the tested substance in a living organism. This is due to the fact that cell cultures are a simplified model in which the tested factor is characterised only at the cellular level and in an artificially maintained microenvironment. However, the interaction of individual organs, systems, substances such as hormones, and the actions of the immune system cannot all be considered [42]. Nevertheless, in vitro cultures are still the first step in pathomechanism decoding and drug testing and are an indispensable tool for the detailed characterisation of cell biology, including skin cells. The present study presumed the issues of research on psoriasis (Ps), testing potential in vitro models that best mimic the skin phenotype arising from this disease. Going through the latest literature reports on the pathogenesis of Ps, including interactions between skin and immune cells, and our own studies in this area, it was possible to use various configurations of cell cultures and the factors necessary to select and test models that most accurately mimic features of Ps skin in the course of this study [17,21,43]. Thus, at this time, two-dimensional (2D) in vitro monocultures of human adult low calcium temperature keratinocytes (HaCaTs), primary keratinocytes (pKCs), and co-cultures of HaCaT and human monocytes (THP-1) were selected.

In general, among 2D in vitro models of Ps, monolayer keratinocyte culture, as well as keratinocyte and immune cell’s co-culture system, have attracted interest for the study of Ps and the development of more effective treatments. A monolayer of keratinocytes, being a critical component of the skin’s immune system, is represented either by cell lines or pKCs. The HaCaT cell line (with mutant p53 and loss of p16ink4a due to hypermethylated *p16* promoter) is a spontaneously immortalised human keratinocyte that is widely used for skin biology studies, including inflammation transmission. Despite the many controversies referring to in vitro studies based on epidermal keratinocytes for relevance to Ps, HaCaT cells have most often been used as cellular models to research disease and evaluate the anti-psoriatic activities of test components [44,45]. HaCaTs represent a cheaper alternative to other cell lines, such as human epidermal keratinocyte 001 (HEK001) and keratinocyte Tr (KERTr). HEK 001 cells do not express keratin 10 (KRT10), indicating their basal-type phenotype, while KERTr lacks major histocompatibility complex class I or II molecules [46]. In contrast to many virally transformed keratinocyte cell lines, HaCaTs can express differentiation-specific gene products, including keratin 1 (KRT1) and KRT10, and differentiation markers, such as involucrin (IVL) and filaggrin (FLG) [47]. Seo et al. found that the gene transcription profile alteration of antimicrobial peptide BD2, encoded by *DEFB4* in response to interferon gamma (IFN-γ), interleukin 4 (IL-4), or IL-17A in HaCaT cells, was consistent with the expression pattern of pKCs [47,48]. Furthermore, the gene transcriptional profile of cornified envelope-associated proteins in HaCaTs was generally different from that in pKCs, suggesting that the HaCaT cell line has limitations as a model to study normal skin barrier development. They might not be as physiologically relevant as pKCs, but they are still easier to obtain and culture. The choice of an in vitro model is dictated by the purpose of the research. The monolayer in vitro model of Ps-like HaCaT has already been demonstrated as a valuable first step model system in the evaluation of nutraceutical agents, such as genistein, for Ps improvement [44,45]. Regarding the co-culture system, skin-specific keratinocytes and immune cells are key participants; responding to stimulation, they serve a very important role in the immunopathogenesis of Ps. The crosstalk between keratinocytes and immune cells is responsible for the induction and maintenance of Ps. To mimic the clinical situation as closely as possible, keratinocytes in mono- and co-cultures need to be stimulated to the Ps phenotype, similar to keratinocytes derived from Ps plates, to maintain their disease characteristics. Notably, Ps keratinocytes are difficult to culture. Initial attempts to generate an in vitro Ps model were performed using skin cells from freshly isolated dermal Ps punches within the lesion. However, the disease phenotype, as assessed by examining the expression of four selected Ps-associated genes (*CAMP*, *DEFB4*, *PI3*, and *TNF-α*), was lost during in vitro cell culture expansion [17,49,50]. Therefore, the addition of definite cytokines was essential to maintain the Ps genes’ signature. However, considering the lack of reproducibility and the difficulties associated with sourcing isolated lesional Ps skin, this approach is inefficient and does not meet the needs of a reliable, accessible, and predictive preclinical model. In the face of such facts, the emphasis, considering the success of a wide range of studies, is on the use of commercial cell lines, as well as keratinocytes taken from fresh skin tissues of healthy people with so-called postoperative rejects (i.e., waste skin material), cultured in vitro and appropriately stimulated to obtain a Ps keratinocyte inflammatory profile. Such a method is beneficial to the broad 3 Rs philosophy and has the potential for the significant “replacement” of live animal experimentation.

The study of Ps inflammation-based modulation in keratinocytes has been detailed in numerous papers with major cytokines, chemokines, and accessory molecule-releasing cells in the viable epidermis. Dysregulation and the abnormal expression of inflammatory mediators in keratinocytes are implicated in the pathogenesis of many skin diseases, such as Ps. In resting keratinocytes, immune mediators are at very low levels; however, under stimulatory conditions, these cells express inflammatory factors that transmit signals to cells of innate and adaptive immunity. A complex cytokine network has been described in Ps, and the central role of pro-inflammatory cytokines produced by epidermal and immune cell infiltration, such as IL-1, IL-17, IL-22, oncostatin M (OSM), and tumour necrosis factor alpha (TNF-α), has been highlighted. Methods to induce inflammation models on cells in vitro using a combination of these five compounds (5MIX) have thus been of interest for a long time. Furthermore, imiquimod (IMQ), serum, and treatment with phorbol 12-myristate 13-acetate (PMA), lipopolysaccharide (LPS), and interferon gamma (IFN-γ) for Ps-like inflammation stimulation, were included in this research. IMQ was chosen in reference to Varma et al. (2017) [35], while the serum was based on Holzmann et al. (1988) [51], Laubscher et al. (1991) [52], and Priestley and Adams (1985) [53]. PMA, a commonly known activator of protein kinase C (PKC) and nuclear factor-kappa B (NF-κB), can induce monocytic THP-1 cells to differentiate into macrophages. This process is preceded by monocyte growth arrest in the G1 phase of the cell cycle. Together with LPS and IFN-γ, PMA was of interest because of previous reports [54,55].

**Figure 3:**
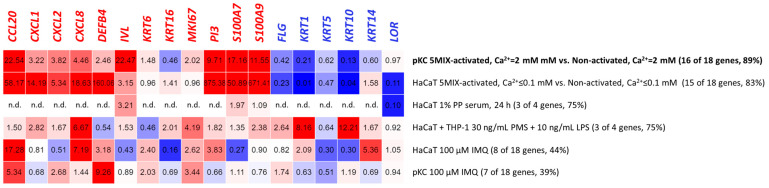
Portraits of all tested in vitro cell models and the model best mimicking the Ps phenotype. Gene names in red are upregulated and in blue are downregulated, according to the literature reports as stated in Table 1. N.d.—no data available.

As we were unable to locate literature reports on the transparent indication of Ps models for determination of Ps inflammation modulation, the key target of delivered therapeutics, we carried out a detailed investigation to this end. To assess an in vitro method to generate the best Ps skin-like inflammatory model, the gene expression profile of Ps skin-specific markers was screened in humans using data obtained from a number of in vitro models mimicking the skin-specific status, with considerable variability being shown. As a result, a total of 18 carefully selected marker genes were included in this study, and among them, there was gene coding for 10 keratinocyte proliferation and differentiation markers (i.e., *IVL*, *FLG*, *KRT1*, *KRT5*, *KRT6*, *KRT10*, *KRT14*, *KRT16*, *LOR*, and *MKI67* genes), four antimicrobial peptides (i.e., *DEFB4*, *PI3*, *S100A7*, and *S100A9* genes), and four chemokines (i.e., *CCL20*, *CXCL1*, *CXCL2*, and *CXCL8* genes. The wide spectrum of data obtained because of our research allowed us to conclude that the in vitro primary cells, i.e., pKC monoculture activated with 5MIX inducers (i.e., IL-1α, IL-17A, IL-22, OSM, and TNF-α in mixture) were preferable at low calcium (Ca^2+^) concentrations in the medium (i.e., below 0.1 mM; Figure 3). This seemed to be the most reliable research model, as a powerful synergy was demonstrated for the Ps expression profile of most of the genes tested (14 or 16 out of 18; i.e., 78 or 89% of analysed transcripts, respectively; Figure 1b), inducing the keratinocyte proliferation rate, poor epidermal differentiation program, and immune-based inflammation response, which were consistent with those in patients with Ps (Table 1).

These results were consistent with our very preliminary analyses made on primary normal human epidermal keratinocytes (NHEK; data not shown). The addition of the cytokine mix (5MIX) at 0.1 mM Ca^2+^ (condition: activated, Ca^2+^ ≤ 0.1 mM) inhibited keratinocyte differentiation, which was consistent with information reported in the literature [56]. Inflammation is strongly associated with morphological changes and impaired differentiation of the epidermis; furthermore, significantly suppressed expression of proliferation/differentiation genes, such as *FLG*, *KRT1*, *KRT5*, *KRT10*, *KRT14*, and *LOR*, was observed, while upregulated transcript levels of *IVL* and *MKI67* were observed in pKCs and, to some extent, HaCaT cells stimulated with 5MIX at 2 mM Ca^2+^ compared to non-activated cells maintained at Ca^2+^ ≤ 0.1 mM or 2 mM medium (Figure 1a). The loss-of-function mutation in the *FLG* gene has been associated with Ps, similar to the *LOR* mutation that alters the differentiation of keratinocytes, with attenuated expression in Ps lesion skin being observed [57,58,59]. Keratin pairs KRT5–KRT14 and KRT1–KRT10 were shown to be directly involved in cell cycle control, which begins keratinocyte differentiation. In addition, their cellular absence or reduction exhibited greater epidermal proliferation [60,61]. The KRT5–KRT14 to KRT1–KRT10 switch during early keratinocyte differentiation was recognised, and follow-up studies revealed altered proliferation and inflammatory features of keratinocytes, contributing to their hyperproliferation and innate immune activation in response to an epidermal barrier breach, followed by the autoimmune activation of T cells that drive Ps. Gene coding for MKI67 proliferation and IVL early differentiation markers were significantly upregulated (Figure 1a,b and Figure 2), which agrees with the Ps pattern (Table 1). Notably, *CXCL1*, *CXCL2*, and *CXCL8* attracting chemokines that interacted with the antigen-presenting cells and lymphocytes in the area of inflammation were significantly enriched after 5MIX combination stimulation at those conditions. Additionally, *CCL20* expression, which contributes to plasmacytoid dendritic cell (pDC) activation and the recruitment of T helper 17 (Th17) cells as a ligand for CC chemokine 6 (CCR6) receptor, was upregulated, resulting in a positive feedback loop with a decisive role in the maintenance of Ps [1]. Our HaCaT and pKC monocultures, activated with the 5MIX inducers at Ca^2+^ ≤ 0.1 mM or 2 mM, also demonstrated a potent synergy on the Ps expression profile of *DEFB4*, *PI3*, *S100A7*, and *S100A9* gene coding for proinflammatory antimicrobial peptides (Figure 1a,b and Figure 2). In our earlier independent studies, a multi-fold increase in *S100A7* and *S100A9* expression was observed in PP vs. NN skin from punch biopsies (our unpublished data, [62]). In general, these barrier alarmin molecules are well known to modify host inflammatory responses by a variety of mechanisms, including the action of angiogenic agents, chemotactic factors, and other cellular regulators implicated in the recruitment of inflammatory infiltrates during Ps development [63]. Their specific molecular mechanisms are as follows: *DEFB4* protein product with chemokine activity recruits memory T cells and immature DCs to the area of microbial invasion through interaction with CCR6; peptidase inhibitor-3 (PI3) produced by epithelial and immune cells with anti-inflammatory properties plays an important role in the inhibition of inflammatory neutrophil derived elastase; S100A7 and S100A9 augment the production of CCL20, CXCL1, CXCL2, and CXCL8 in keratinocytes [1,14,64].

Moreover, the gene expression pattern in GEO profiles of Ps patients, when referring to the seven datasets for skin biopsies (i.e., GDS2518, GDS3539, GDS4600, GDS4602, GDS4606, GDS5392, and GDS5420 microarray datasets), was consistent with the suspected increased expression characteristic for the Ps phenotype, which in turn was not that reliable for in vitro cell culture of keratinocytes, when referring to the six datasets (i.e., GDS2081, GDS2611, GDS2732, GDS3011, GDS4598, and GDS4599 microarray datasets; information from Table 1 in comparison to the data in Figure 2). This, in turn, indicates the significance of the results documented in this report and justifies the undertaken research.

Overall, the aim of this study was to establish an in vitro inflammatory Ps model, recapitulating specific characteristics of lesional Ps skin and providing a reliable opportunity to discover the still unknown mechanism of Ps disease, and to develop a protocol for screening new therapeutic compounds.

## 5. Conclusions

The methods outlined here draw on several scientific approaches, making use of skin waste biological tissues collected with the consent of the donor as a means of determining Ps skin inflammation and focusing on probing aspects of inflammation caused by this type of dermatosis. Among the various factors tested, a reproducible procedure that yielded an in vitro Ps-like inflammatory model was defined, using the pKC monoculture treated with a cytokine cocktail (5MIX) at a calcium (Ca^2+^) concentration (i.e., 2 mM) in an applied medium and exhibiting the Ps-specific marker gene profile. For further study of the model selected and portrayed in this report, it may be interesting to continue complementary research using, for example, confocal microscopy and other advanced methods. pKCs are generally accessible, inexpensive, and free of ethical restrictions. Based on waste skin material, the method is conducive to the broad “3 Rs” philosophy and has the potential for the significant “replacement” of live animal experimentation. Thus, a review of in vitro Ps models being tested to select the optimal one for studies on Ps was provided.

## Figures and Tables

**Table 1:** Gene markers designated for the establishment of in vitro psoriasis (Ps)-like inflammation models with cytokine mix (5MIX)-, imiquimod (IMQ)-, and serum-induced monoculture of HaCaT or pKC keratinocytes, and phorbol 12-myristate 13-acetate (PMA), lipopolysaccharide (LPS), and interferon gamma (IFN-γ) co-culture of HaCaT and THP-1 cells.

Functionality Clusters	Gene Symbol	Effector Protein	Gene Expression in Ps Phenotype Referring to the Literature Data ↑ (Upregulation) or ↓ (Downregulation)
Keratinocyte differentiation markers	*IVL*	Involucrin	↑ Ishida-Yamamoto et al., 1995 [22]
	*FLG*	Profilaggrin	↓ Bowcock et al., 2001 [23]
	*KRT1*	Cytokeratin 1	↓ Elango et al., 2018 [24]
	*KRT5*	Cytokeratin 5	↓ Cheng et al., 2018 [25]
	*KRT6*	Cytokeratin 6	↑ Thewes et al., 1991 [26]
	*KRT10*	Cytokeratin 10	↓ Thewes et al., 1991 [26]
	*KRT14*	Cytokeratin 14	↓ Thewes et al., 1991 [26]
	*KRT16*	Cytokeratin 16	↑ Thewes et al., 1991 [26]
	*LOR*	Loricrin	↓ Giardina et al., 2004 [27]
Antimicrobial peptides	*DEFB4*	β-defensin	↑ Hollox et al., 2008 [28]
	*PI3*	Peptidase Inhibitor 3 (SKALP)	↑ Schalkwijk et al., 1993 [29]
	*S100A7*	S100 Calcium Binding Protein A7	↑ Madsen et al., 1991 [30]
	*S100A9*	S100 Calcium Binding Protein A9	↑ Madsen et al., 1991 [30]
Chemokines	*CCL20*	C-C Motif Chemokine Ligand 20	↑ Harper et al., 2009 [31]
	*CXCL1*	C-X-C Motif Chemokine Ligand 1	↑ Suárez-Fariñas et al., 2012 [32]
	*CXCL2*	C-X-C Motif Chemokine Ligand 2	↑ Kennedy-Crispin et al., 2012 [33]
	*CXCL8*	C-X-C Motif Chemokine Ligand 8	↑ Suárez-Fariñas at al., 2012 [32]
Proliferation marker	*MKI67*	Marker Of Proliferation Ki-67	↑ De Mare et al., 1990 [34]
